# Supplementary material for: An intriguing new diapsid reptile with evidence of mandibulo-dental pathology from the early Permian of Oklahoma revealed by neutron tomography
Source: PLoS One. 2022 Nov 30;17(11):e0276772. doi: 10.1371/journal.pone.0276772 (PMC9710763; doi:10.1371/journal.pone.0276772)
Supplement: Attachment — Submitted filename: Reviewer Notes.docx [file pone.0276772.s001.docx]

Attached here are the responses to the reviewers, in red.

Reviewer #1: PLOS ONE Review

Manuscript Number: PONE-D-22-17394
Title: An intriguing new diapsid reptile with evidence of mandibulo-dental pathology from the early Permian of Oklahoma revealed by neutron tomography
The authors have presented a detailed description of new material from the Richards Spur locale consisting principally of mandibular and palatal elements, but also some cranial material, including an incomplete jugal and maxilla. They conclude that the holotype and 4 referred specimens are attributable to a new genus and species of diapsid reptile (Maiothisavros diane), based on the likely presence of a lower temporal fenestra, a suborbital opening and the morphology of the maxillary canal. They also recognise some pathology of the right dentary in one referred specimen. They further conclude that histological analysis of the marginal dentition suggests ‘pleurodont’ tooth implantation and a relatively slow rate of tooth replacement.
As the authors suggest, diapsid material from the Late Carboniferous and Lower Permian is very rare and any addition to our knowledge of early reptile diversity and evolution is welcome. For this reason, I fully support the publication of this article. However, there are several revisions and amendments that I propose the authors consider prior to publication, which are detailed below.

Abstract (lines 25-26). “Notwithstanding the hypothesis that varanopids are diapsids rather than synapsids…”. The authors are right to mention this alternative hypothesis, but I note it is restricted to the abstract only and omitted from the main text. I think it appropriate to include this in the main text, especially since the text implicitly follows the ‘varanopids as synapsids’ hypothesis. Done

Abstract (Lines 42-43) “…pleurodont implanted dentition reminiscent of extant snakes…” – please refer to comments in ‘Tooth attachment and Implantation (lines 490 – 492)’ below. removed “reminiscent of extant snakes”

Introduction (line 51) “…now dated 316M.” The age of the Joggins material has certainly been subject to some debate. Benton et al. (2015) have suggested 318Ma, based on comparisons with the Langsettian European time unit. However, authors may have a more recent assessment, in which case a reference would be appropriate. Also ‘316 M’ should read ‘316 Ma’. Corrected 316 M to 318 Ma.

Rygel, M. C., Lally, C., Gibling, M., Ielpi, A., Calder, J. H. & Bashforth, A. R. (2015). Sedimentology and stratigraphy of the type section of the Pennsylvanian Boss Point Formation, Joggins Fossil Cliffs, Nova Scotia, Canada. Atlantic Geology, 51, 1–43.

Introduction (line 52) “…Protoclepydrops…” – should read “Protoclepsydrops”. Also, there is some ongoing discussion on the affinities of the material assigned to this taxon - see Mann and Patterson (2020). done

Introduction (lines 52-54, sentence ending “…in the fossil record.” The sentence doesn’t read well. Perhaps substituting ‘however’ for ‘but’ in line 54? done

Introduction (lines 57-58, sentence ending “…turtles and squamates.” This implies that both turtles and squamates only began to diversify after the end Cretaceous extinction, whereas certainly squamates had already undergone significant diversification by the end of the Cretaceous (see Ricklefs et al. 2007). Changed to clarify

Introduction (line 60,) sentence commencing Although amniotes synapsids….”. I suggest a small revision – perhaps “Although amniotes, in particular synapsids, parareptiles etc…”. done, sentence removed.

Histology (lines 141 – 142). Repetition of “…prior to histological analysis.”. removed second repetition of “prior to histological analysis”

Locality (line 191). Th date range 289-286 Ma should be described as Artinskian age (see International Chronostratigraphic Chart v.2022/02). done

Diagnosis (Lines 198-199) “…. differs from varanopids in lacking any lateral suborbital ornamentation.” Whilst this statement correctly reflects the condition found in derived varanopids, jugal ornamentation does not occur in the ‘basal’ varanopids Archaeovenator (Reisz and Dilkes 2003) and Ascendonanus (Spindler et al. 2018), both of which possess slender triradiate jugals with narrow suborbital and subtemporal rami. Replaced “varanopids” with “*mycterosaurine* and *varanodontine* varanopids”

Skull Roof (line 227) “…2 in-situ teeth”. It is difficult to determine from figure 1B that these two teeth can be confidently described as ‘in-situ’, since no trace of the underlying premaxilla remains. Therefore, the assessment of the number of teeth in the premaxilla is likely to be somewhat speculative. Changed phrasing to “ 2 seemingly in-situ teeth”

Skull Roof (line 242). Delete “...a...”. done

Skull Roof (line 297) “The subtemporal or posterior process…”. This ramus of the jugal has already been introduced in line 293. So perhaps delete “...or posterior…”? deleted “or posterior”

Skull Roof (line 299). The jugal in the varanopids Archaeovenator and Ascendonanus are very similar in shape to the jugal in ROMVP 87366. So, it is a little misleading to imply all varanopids possess a more robust process. **Disagree.** Careful examination of those two varanopids indicates a generally more robust posterior subtemporal process than in this taxon. Nevertheless, we changed the wording slightly.

Skull Roof (lines 300 – 301) “…lacks the kind of rugosity seen in varanopids…”. Refer to note under ‘Diagnosis’ above. Perhaps amend to “some varanopids”. Added “some

Fig.5. It is difficult to distinguish between the cultiform process and the pterygoid, certainly in image A. Perhaps a more distinctive colour can be used for the cultriform process? Disagree, the cultriform process is a dark vibrant orange color while the pterygoid is a soft mellow orange color and are readily distinguishable with the additional aid of labels.

Palate (line 315). It appears from Fig.5 that these might be better described as ‘fields’ rather than ‘rows’, since the teeth are arranged randomly within each distinctive field. This logic is applied in line 317. The teeth on the posterior margin of the transverse process are indeed a ‘row’ of single teeth. done

Palate (lines 335 – 336). It would be helpful to show the possible ectopterygoid in a different colour scheme or at the very least identify it in the figure. No ectopterygoid actually present

Palate (lines 339 – 341) sentence beginning “The ventral surface…”. It would be useful for a call-out to indicate which of the 4 images of Fig.6 reflect the elements described in the text and perhaps a label as well (i.e., the margin of the internal naris, the lateral process indicating the maxillary ramus of the palatine etc.). The latter is important since the presence of a suborbital opening (an important diagnostic feature in determining the diapsid affinities of this taxon) should be evidenced as clearly as possible. Comment is referring to Fig 5. Margin of the internal naris is not preserved, so cannot be labeled. Margin of the suborbital fenestra is labeled and call outs have been added.

Mandible – general. Again, the text lacks specific call-outs for each of the 4 images of Fig.6 with respect to important and distinctive morphological observations (e.g., the anteroventral expansion of the dentary). done

Mandible (lines 388 and 404). Suggest this should read “…other eureptiles and parareptiles.” Since it currently implies the taxon is a parareptile. Since this is also an unusual morphology among synapsids, perhaps “…other early amniotes.” may fit better. Changed to “eureptiles and parareptiles”

Mandible (line 417) “…. displays pleurodont dentition”. Refer to comments in ‘Tooth attachment and Implantation (lines 490 – 492)’ below.

Mandible, paragraph commencing “Each dentary has…”. In general, there should be more call-outs to figures, particularly when discussing tooth positions. done

Mandible, paragraph commencing “The splenial is…”. Please provide more specific call-outs to individual images in Fig. 6 when describing anatomical detail. done

Mandible, line 447. Do the authors mean to say “splenial symphysis” or would “mandibular symphysis” be more accurate? Changed to “mandibular symphysis”

Mandible (line 450). Should this read “the internal or lateral surface contains the same large foramen as the medial surface….”? Changed to “the internal or medial surface contains the same large foramen as the lateral surface”

Tooth attachment and Implantation (lines 490 – 492). I agree that tooth attachment appears to be ankylosed to the dentary via the mineralization of periodontal tissue. In addition, the dentary is higher labially than lingually. However, this does not necessarily indicate pleurodonty sensu stricto. In Bertin et al. (2018) – the reference applied in the text – ankylosis to the dentary via mineralized attachment tissue, with no periodontal space, is associated with subthecodont implantation (see Bertin et al. Fig. 2B). Bertin et al. define subthecodonty as implantation in an asymmetrical shallow socket, (Fig.3E), rather than a pleurodont implantation where there is a distinct gradient between the labial and lingual margins (Fig. 3C). Text Fig. 9B is more reflective of a subthecodont condition as defined by Bertin et al., (although I have heard such implantation described as subthecodont and subpleurodont). In any event, the similarity of this implantation to snakes (as suggested in the abstract) is questionable. Removed reference to snakes. Replaced “pleurodonty” with “subthecodonty”. Replaced “not within a deep socket” with “with attachment in an asymmetric shallow socket”. Replaced reference to pleurodonty with subthecodonty throughout manuscript.

Diagnostic Morphologies (lines 547 – 548). The flattened ventral surface had already been noted in lines 544 – 545, so this seems a little repetitive. removed

Diagnostic Morphologies. It is interesting to note that the authors have omitted to mention here the presence of both an anterior and posterior coronoid in Maiothisavros diane. This is an important feature of early amniote phylogeny. Among the majority of early diapsids, indeed early sauropsids in general, there is only a single coronoid. Two coronoids have only been reported in Hylonomus (Carroll 1964, although somewhat contentious) and Araeoscelis (Vaughn 1955). Indeed, the similarity with the latter taxon might suggest an alternative hypothesis to the possible neodiapsid affinities of M. diane. So, the clear identification of two coronoids in Maiothisavros diane is a key anatomical observation. Two, or even three, coronoids are present in stem-amniotes. Generally, two coronoids are present in synapsids. Only one is present in the varanopids Archaeovenator and Mesenosaurus. The implications of two coronoids in M. diane is therefore certainly worthy of reference.
 The two coronoids have been added to the diagnosis. Its an interesting phenomenon but two coronoids are found in quite a few synapsids, but not all, and in parareptiles but not captorhinids, so the retention of the second coronoid may not be as significant to the phylogenic implications of this new taxon.

Supporting evidence for placement in Diapsida (lines 572 – 573) “…varanopid synapsids, in lacking any lateral ornamentation….”. Please refer to comments in ‘Diagnosis (Lines 198-199)’ above. Addressed in previous comment.

Supporting evidence for placement in Diapsida (lines 576 – 577) “…hallmark feature...”. If the authors consider this a synapomorphy of diapsid reptiles, perhaps this would be a more appropriate wording. done

Supporting evidence for placement in Diapsida (lines 576 – 577), sentence commencing “Interestingly, the morphology….”. This sentence is a little confusing. The morphology of the anterior medial foramen is not the issue, but rather the shape of the maxillary canal directly ventral to it. Changed to clarify

Supporting evidence for placement in Diapsida (lines 588 – 590), sentence commencing “However, the similar morphology….”. It is not clear what the authors are suggesting here. If they infer that there may be a closer phylogenetic affiliation between M. diane and archosauromorphs, I should advise caution. At least until it can be tested for congruence within a phylogenetic framework. Rephrased to be more cautious.

Comparison with Orovenator mayorum (line 604) “…varanopid condition of lateral suborbital rugosities…”. Please refer to comments in ‘Diagnosis (Lines 198-199)’ above. addressed in previous comment.

Comparison with Orovenator mayorum (line 609) “Interestingly…”. Perhaps a little too repetitive given the same adjective is used in line 606. “interestingly” used in line 606 was removed.

Comparison with Orovenator mayorum (line 614) “The sub orbital process is underlain by the maxilla in O. mayorum…”. The authors do not follow this statement with a comparison for M. diane, so it is somewhat redundant. Also, “O. mayorum” is not italicized. This sentence was reworded to “The posterior process of *O. mayorum*, is also very delicate in contrast to the more robust bifurcate condition of *M. dianeae*.”

Histology (line 639) “…pleurodont dentition…”. Please refer to notes under ‘Tooth attachment and Implantation (lines 490 – 492)’ above. done

Histology (line 641). “…it…” should read “...is...” and “…normal…” should read “…normally…” done

Conclusion (lines 699 – 671), partial sentence commencing “We propose that…” and ending “…in younginiforms.” I cannot agree that the number of autapomorphies in any given taxon provides an indication of a more crown-ward phylogenetic position. Perhaps this phrase is used by the authors in respect to lines 588 -590 (see above). In which case I would continue to advise caution. Changed to reflect a more cautious approach

Conclusion (lines 673 – 675) sentence commencing “In conclusion…”. This sentence basically repeats the sentence commencing on line 667 in the concluding paragraph. Deleted sentence commencing on line 667 and replaced it with sentence formally in line 673 – 675 because they essentially stated the same thing.

Dr. David Ford

5/7/22

References:

Benton, Michael J., Donoghue, Philip C.J., Asher, Robert J., Friedman, Matt, Near, Thomas J., and Vinther, Jakob. 2015. Constraints on the timescale of animal evolutionary history. Palaeontologia Electronica 18.1.1FC; 1-106. <https://doi.org/10.26879/424>

Bertin, T.J., Thivichon-Prince, B., LeBlanc, A.R., Caldwell, M.W. and Viriot, L., 2018. Current perspectives on tooth implantation, attachment, and replacement in Amniota. Frontiers in physiology, 9, p.1630.

Carroll, R.L., 1964. The earliest reptiles. Zoological Journal of the Linnean Society, 45(304), pp.61-83.

Mann, A., and Paterson, R.S., 2020. Cranial osteology and systematics of the enigmatic early ‘sail-backed’ synapsid Echinerpeton intermedium Reisz, 1972, and a review of the earliest ‘pelycosaurs’. Journal of Systematic Palaeontology, 18(6), pp.529-539.

Ricklefs, R.E., Losos, J.B. and Townsend, T.M., 2007. Evolutionary diversification of clades of squamate reptiles. Journal of evolutionary biology, 20(5), pp.1751-1762.

Reisz, R.R. and Dilkes, D.W., 2003. Archaeovenator hamiltonensis, a new varanopid (Synapsida: Eupelycosauria) from the Upper Carboniferous of Kansas. Canadian Journal of Earth Sciences, 40(4), pp.667-678.

Spindler, F., Werneburg, R., Schneider, J.W., Luthardt, L., Annacker, V. and Rößler, R., 2018. First arboreal' pelycosaurs'(Synapsida: Varanopidae) from the early Permian Chemnitz Fossil Lagerstätte, SE Germany, with a review of varanopid phylogeny. PalZ, 92(2), pp.315-364.
Vaughn, P. P. 1955. The Permian reptile Araeoscelis re-studied. Harvard Museum of Comparative Zoology, Bulletin 113:305-467.

Reviewer #2: The authors describe a partial skull and mandible from the lower Permian Richards Spur locality of Oklahoma, U.S.A., as a new genus and species of diapsid reptile. I have no problem with the identification of the specimen as a diapsid reptile, and I do agree that the material is too limited to justify a new phylogenetic analysis, nor do I expect the authors to resolve the question of varanopids-as-diapsids at this time on the basis of such paltry material. However, I do not think that the new diapsid is as unique as the authors claim. For instance, the authors interpret the tooth implantation as pleurodont, but that interpretation is at odds with recent histological work on pleurodont squamate teeth. The authors also describe the dention as “snake-like” but the only teeth that look snake-like to me are the maxillary caniniforms and those forming the caniniform region in the dentary (it is hard to tell for the premaxillary teeth); the remaining teeth are stout with recurved tips but are not remarkably snake-like. Removed reference to snakes

MAJOR COMMENTS

The authors suggest that the new diapsid may be a neodiapsid, but the only character that supports a neodiapsid affinity is the slender subtemporal process of jugal. The maxillae exhibit caniniform teeth (although the authors refer to these teeth only as “larger teeth”—however the pulp cavities of these teeth look to be the same size as the teeth immediately preceeding and succeeding the caniniform teeth!), unlike Paleozoic neodiapsids, and the lower temporal fenestra is closed (the jugal exhibits sutural surface for the quadratojugal).

I disagree that tooth implantation is pleurodont. The histological sections provided by the authors indicate that the labial and lingual dentine walls are the same length and thus symmetrical. This is unlike the pleurodont teeth described by LeBlanc et al. (2020) for iguanids. In figure 9b the tooth sits in a shallow socket and implantation appears to be subthecodont; i.e. the jaw bone exhibits no pleura.

General: where authors state bone X forms a suture with bone Y, I think it would be appropriate to identify the kind of suture, e.g. “the angular forms overlapping sutures with the dentary and the splenial” or “the jugal appears to have formed a scarf joint with the quadratojugal”.

OTHER COMMENTS:

Line 36: change “while” (connotes time) to “whereas” (comparative). See also lines 241, 309, 332, 336, and 642. Done other “while” word choices were also changed to “whereas” where appropriate.

Line 55: change “Diapsids represents” to “Diapsids represent” done

Line 60: change “Although amniotes synapsids, parareptiles, and non-diapsid eureptiles” to “Although synapsids, parareptiles, and non-diapsid eureptiles” done, word choice addressed in previous reviewer #1 comment.

Lines 42-45: change these lines to “Lake Shale of Kansas [3], already was characterized by the presence of a suborbital fenestra, bound by the palatine, ectopterygoid, and maxilla [4], and two temporal fenestrae, consisting of a lower or inferior temporal fenestra bound by the jugal, quadratojugal, postorbital, and squamosal, and an upper or superior temporal fenestra bound by the postorbital, parietal, and squamosal.” [note addition of commas] done

Line 76: change “Early Permian” to “early Permian” done

Line 87: change “Reisz, et al (2011a)” to “Reisz et al. [5]” done

Line 115: is the matrix of the specimen limestone? I thought it would be clay or marlstone. Added “the largely limestone matrix”

Line 171: I think the new species name should follow line 170 here as “Maiothisavros diane gen. et sp. nov.” otherwise the following section (Etymology) makes no sense. done

Line 177: if you are honouring Diane with a specific epithet then it should be “dianeae” (-ae suffix). If you intend to keep the specific epithet as “diane” you should declare it a noun in apposition. done

Line 181: change “mandibles” to “mandible” [note that each gnathostome has a single mandible that can be subdivided into left and right mandibular rami] done

Lines 194-199: the presence of an anterior coronoid is rare among Paleozoic reptiles in general and in diapsids in particular, and should be included in the Diagnosis added “

Line 203: change “mandibles” to “mandible” done

Line 214: with regards to “mediolateral thickness of the existing dentition” do you mean “labiolingual thickness of the existing dentition” ? changed “mediolateral” to “labiolingual”

Line 215: change “a capture and kill predatory behavior” to “a capture-and-kill predatory behavior” done

Line 230: with regards to “mediolateral” here I think you should be using dental terminology such as labial, ligual, distal, and mesial for orientation. See also line 243. Replaced “mediolateral” with “labiolingual”

Line 233: “width” is not a helpful term when describing teeth: do you mean labiolingual basal diameter or mesiodistal basal diameter ? done

Lines 230-231: with regards to “The premaxillary teeth appear to lack serrations on both carinae of the tooth crown” I don’t see any carinae on those teeth in figure 1. If indeed present, please describe the carinae and then mention that they lack serrations. Added more description by adding “the medially trending”

Lines 236-238: describe the surficial texture of the premaxillary teeth here and then describe the surficial texture of the anterior dentary and maxillary dentition as consistent with that found in the premaxillary dentition. done

Line 247: the parenthatical statement “vertical fluting to form an effective piercing edge” is interpretive and belongs not in the desrciption section but the discussion section. Also, note that “fluting” actually comprises long narrow furrows with rounded bottoms, whereas the term “reeding” is used for (more or less) parallel ridges with rounded surfaces (e.g. google “Fluted vs Reeded” for visual examples). the ridges on the teeth shown in figure 2 are better described as “reeding” to me. Removed “vertical fluting to form an effective piercing edge”. Replaced fluted terminology with reeded terminology and added short description of reeding “long parallel ridges with rounded surfaces”

Line 260: explanation for abbreviation “c” (carina [?]) missing. Added “c, carina;” to figure description

Line 267: change “tooth position mx05, mx08, mx10, mx14” to “tooth positions 5, 8, 10, and 14”, Do similar for “mx15 or mx16” in next line and line 270. Label these tooth numbers on figure 3. Adjusted to “6, 9, 11, 14” and deleted “mx16” as branching points of the foramina and changed left maxilla foramina branching points to “8, 9, 10, 11, and 13”

Lines 282-283: NOTE THAT the left maxilla (tooth count of 13) is shown in parts A and B and the right maxilla (tooth count of 24) is shown in parts C and D (the reverse is indicated in the caption). NOTE THAT part A and C are lateral views and parts B and D are dorsal views. Also, please rotate image in part D 180 degrees to facilitate comparison (lining up) with C. done

Lines 285-288: change these lines to “Although incomplete, the right jugal (Fig 4) exhibits the triradiate shape typical of early diapsids. The dorsal process is complete and displays a characteristic flat spoon shape up to the tip where the anterior surface would suture to the postorbital bone.” done

Line 297: typo in “subtermporal” done

Line 304: change “than in the neodiapsid” to “that of the neodiapsid” done

Line 314: change “while the left pterygoid preserves only its central palatal portion” to “whereas only the central palatal portion of the left pterygoid is preserved.” done

Line 317: change “lateral most” to “lateral-most” done

Line 323: “but to a smaller degree” [?] removed “but to a smaller degree” and added “minor curvature”

Line 330: change “shelves” to “low mounds” or “swellings” [?] replaced “shelves” with “swellings”

Line 336: please label the ectopterygoid in figure 5 given that it is “not shown as a separate element in segmentation” No ectopterygoid actually present

Lines 338-347: if only “the medial anterior portion of the left palatine is present” how does the “lateral process indicate “the presence of a rather large suborbital fenestra” ? labeled in Figure 5. Added “The lateral process is robust, and the size of the suborbital fenestra posterior to it is therefore wide and long”

Line 349: the cultriform process is trough shaped and only “U-shaped” in cross section. Replaced“U-shaped” with “trough shaped”

Line 353: change “foramen” to “foramina”. done

Line 362: change “right mandible” to “right ramus” done

Line 363: change “left mandible” to “left ramus” done

Line 366: delete “elements” done

Line 404: change “other parareptiles or eureptiles” to “other eureptiles or in parareptiles” done

Line 415: capitalize “carboniferous” done

Lines 418-423: change the tooth position “nomenclature” from “d01 to d03” to “1 to 3”, etc. in this paragraph. Do the same for lines 440 and 511.

Lines 419-420: with regards to “with the largest teeth observed in mid-section (d05 to d08)” does not make sense if there are 21 dentary teeth; the “mid-section: would be, if anything, comprised of dentary teeth 8 to 12. It looks to me that dentary teeth 5 to 8 form a caniniform region. Removed “mid-section” and replaced with “positions 5 to 8”

Lines 285-XXX: change these lines to “ROMVP 87367, an additional fragmentary right dentary belonging to another individual of Maiothisavros diane, was also segmented (Fig 8).” done

Line 441: change “Abbreviations:” to “Abbreviation:” done

Lines 448-450: Malcolm Heaton (1979) identified this foramen as the foramen intermandibularis oralis. You can see it quite clearly in the left splenial in figure 6b (hint: you should label it). Finally, reword the sentence beginning “The internal or lateral surface contains the same single large foramen. . .” replaced “has a single large foramen that forms a small groove in the medial or lingual surface in the proximity of the foramen. The internal or medial surface contains the same single large foramen as the lateral surface and has a limited anteriorly extending groove positioned in the anterior portion of the splenial” with “Both right and left splenial also clearly display the intermandibularis oralis”

Line 479: change “left mandible” to “left mandibular ramus” done

Line 534: change “Abbreviations:” to “Abbreviation:” done


Line 539: change “early Permian” to “lower Permian” done

Line 589: typo in “phylogenic” done

Line 593: change “synapsids canal has” to “canals in these synapsids have” done

Line 614: close up “sub orbital” and italicize “O. mayorum” done deleted “suborbital” in previous comment

Line 634: what is the base of the tooth? Is this a term used in lieu of root because the authors regard the implantation to be pleurodont? It looks to me that the plicidentine folds are not exposed because they are covered by bone of attachment, as in Captorhinus aguti (see de Ricqles & Bolt 1983). I agree. We have replaced the wording

Line 636: what is “lamalle” ? Lamella? Lamellae? Replaced “lamalle” with “lamellae”

Line 644: change “Mosaurus” to “Mosasaurus” [?] and italicize done

Line 693: italicize “Petrolacosaurus” done

Line 699: change “Ford, & Benson, R. B. J.” to “Ford, D. P., Benson, R. B. J.” and italicize “Orovenator mayorum” done

Line 702: change “Debraga” to “deBraga” done

Line 717: change “Belly” to “Bally” done

Line 731: italicize “Alligator” done

Line 733: italicize “Anguis fragilis” done

Line 742: italicize “Deloyhynchus” done

Line 756: italicize “Dinilysia” done

Figure 5: label the margin of the palatine that contributes to the suborbital fenestra. This is critical for the identification of the specimen as diapsid. done

REFERENCE

LeBlanc, A.R.H., et al. 2020. Tooth attachment and pleurodont implantation in lizards: Histology, development, and evolution. J. of Anatomy 238:1156-1178.

Reviewer #3: OVERALL COMMENTS -

This manuscript describes an exceptional new taxon of amniote from the Richard's Spur locality of Oklahoma. The authors argue, I think accurately, that the new taxon is a member of Diapsida. My review comments are restricted to a general issue with some of the specifics of the phylogenetic discussion and individual comments on the description. Numerous times in the text, the authors suggest that the new taxon may not only be a diapsid, but a neodiapsid, a more inclusive group of animals more crown-ward relative to Araeoscelidea. To further explore that concept, I think the paper would be aided by the inclusion of the new taxon in a phylogenetic study of early amniotes. Problematically, an analyitical framework with the range of taxa required to asssess this taxon are very limited. I might suggest the following:
The original analysis used to assess Orovenator mayorum by Reisz et al. (2011). Although limited in species diversity, it does properly capture the character transitions described in the paper that support the position of the new taxon as a diapsid and possibly a neodiapsid.
The analysis of Ford et al. (2020) exploring the phylogeny of Amniota. Although the recovery of Araeoscelidia as well removed from Neodiapsid remains controversial, including among authors on this submissison, it may be interesting to see how the new taxon is recovered here.
The analysis of Schoch and Sues (2018) examining the phylogenetic position of the early lepidosauromorph Fraxinisaura. Although that taxon is well removed from the animal described in this submission, the phylogenetic matrix covers a wide range of early amniotes, ranging from captorhinomorphs to “pelycosaurs” to early diapsids.
I used this analysis, and the result are not informative beyond the placement of our taxon within Neodiapsida, rather than with araescelids.
I present my individual comments on the paper below. The figures are all excellent and capture the morphology described in the text well. I have only a few suggestions for additional call-outs in the comments below.

LINE-BY-LINE EDITS -

Page 1
Line 27 – Are you intending the “both lower and upper temporal fenestrae” to refer to all the araeoscelideans and neodiapsid? It is confusing as stated, and troublesome considering the closed fenestra in Araeoscelis and the unknown conditions in more incomplete taxa. Removed wording

Page 3
Line 53 - Seems like these two sentences should be divided. Both points are valid, but do not flow together. reworded sentence to read “Carboniferous, when amniotes represented by various eureptiles, parareptiles, and most notably numerous synapsids diversified rapidly, however diapsids remained very rare in the fossil record”
Line 55 – represent done
Line 56 – Recommend citation of Sues' recent book "The Rise of REptiles" or Carroll's "Rise of Amphibians." Basically any book titled "the Rise of..." done
Line 58 – Birds and crocodylians should receive mention here as well. Mentioned in the previous sentence
Lines 61-62 - I would emphasize this refers primarily to species-level diversity rather than morphological disparity, unless you mean the latter or both. Done

Page 4
Line 69 – Cite a bunch of younginiform, weigeltisaurid papers here. Done, Added references listed below.

YOUNGINIFORMS

Broom, R. 1914. A new thecodont reptile. Proceedings of the Zoological Society of London, pp. 1072–1077.

Carroll, R.L. 1981. Plesiosaur ancestors from the Upper Permian of Madagascar. Philosophical Transactions of the Royal Society of London, **293**(1066): 315–383.

Currie, P. 1980. A new younginid (Reptilia: Eosuchia) from the Upper Permian of Madagascar. Canadian Journal of Earth Sciences, **17**(4): 500–511.

Pritchard, A.C., Sues, H.D., Scott, D., Reisz, R.R. Osteology, relationships and functional morphology of *Weigeltisaurus jaekeli* (Diapsida, Weigeltisauridae) based on a complete skeleton from the Upper Permian Kupferschiefer of Germany. PeerJ. 2021;9:e11413

Gow, C.E. The morphology and relationships of *Youngina* *capensis* Broom and *Prolacerta* *broomi* Parrington. Palaeontologia Africana. 1975;18:89-131.

WEIGELTISAURIDS

Piveteau, J. 1926. Paléontologie de Madagascar, XIII — Amphibiens et reptiles Permiens. Annales de Paléontologie, **15**: 55–178.

Bulanov, V.V., Sennikov, A.G. The first gliding reptiles from the upper Permian of Russia. Paleontol. J. 2006;40:S567-S570.

Page 5
Line 101 – I think you should note that neutron tomography was used here. Quite important to distinguish from usual microCT. done

Page 9
Lines 194-199 - Should the fluted tooth crowns be noted here as well? That does not seem present in other Palaeozoic diapsids. Adds to a good combination of characters. **Added to diagnosis.**

Page 10
Line 213 – slender relative to a specific group of other diapsids or general amniotes? **In general**
Line 215 – a citation to extant predators using this approach would support the statement. **Done,** Benton M. J. Vertebrate palaeontology. 3rd ed. Oxford (UK). Blackwell Science; 2005.

Line 216 – Killed how? Removed “, where the prey was seized and killed almost immediately”
Line 228 - "single space" is vague here. Are you referring to a space equivalent to the mesiodistal width of the other premax teeth? Changed “The single space between the two teeth suggests at least three teeth would have been present on the premaxilla.” to “The single space between the two teeth is mesiodistally equivalent to the other premaxilla teeth, suggesting at least three teeth would have been present on the premaxilla.”
Line 229 - More than what? Deleted “more” added “labiolingually”
Line 230 - Personal preference, but "labiolingually" is commonly used for tooth dimensions. done

Page 11
Line 232 - posteriormost or distalmost. Changed “most posterior” to “posterior most”
Line 233 - "conforms" might be better. "Fits" calls to mind the association of the upper and lower teeth or bones. done
Line 234 - A bit vague. The base of the crown? Root? changed “its” to “the crown”
Line 237 - As this is the first major reference to the crown texture, a description (at least a brief one) would be worthwhile for readers. Added “…, in that they are concial with a labiolingually wide base, slightly recurved, display mesial and distal carinae, and possess plicidentine suroudning the dentine base.”
Line 239 - narrow and elongated in which axes? Sorry, I'm really picky on these descriptors. Added “mediolaterally”
Line 241 – Broken. Determined because of cracks on the elements? How do you know? Changed “Nearly the entire dorsal portion of both maxillae is missing.” To “nearly the entire dorsal portion of both maxillae are visibly missing, as evident by the fractured dorsal surface.”
Line 244 – Reference that your argument in favor of the presence of plicidentine will be provided below in the tooth section. done
Line 245 - Indicate which tooth is the largest. It is a pretty common early amniote/diapsid thing to have some bigger "caniniform" teeth towards the front of the max. This is not present in later neodiapsids like Youngina and Claudiosaurus. Added “large caniniform teeth 6 and 7”
Line 246 - Any chance this groove could be indicated in Figure 2? This description is referring to the reeding of the plicidentine and is indicated on the figure.
Line 250 - Could this also be a consequence of the larger root of the largest maxillary tooth? **Not sure I know where this is in the text. No evidence for that, we did not comment.**

Page 12
Line 253 – Indicate both of these in the figure. The suborbital contribution is especially hard to interpret as-is. The maxillae are incomplete so such labels are unnecessary.
Line 265 - the long axis of the maxilla? Added “long axis of the”

Page 13
Line 286 - To be more explicit, "anteriorly concave spoon-like shape?" yes, done
Lines 292-293 - Somewhat vague about the jugal anterior process. Perhaps "is broken anteriorly." Added “broken anteriorly.” Removed “the least complete ramus of the jugal.”

Page 14 -
Line 316-317 – The size of these pterygoid TP teeth is an interesting character that might be added to the diagnosis. Not sure how widely distributed transverse process teeth on that scale are in Diapsida. **Changed and explained the unusually large size of the tr. Teeth.**

Page 15 -
Line 336 - You might note the absence of such ectopterygoid teeth among neodiapsids. **No ectopterygoid actually present in specimen**

Page 16 -
Line 380 - They're quite separate phylogenetically, but you might make comparisons with Tanystropheus and Azendohsaurus, regarding the expanded symphyseal region. agreed, similarity is mentioned.

Page 17 -
Line 390 - Perceived homology with the Meckelian groove? Added “Meckelian”
Lines 405-406 - A condition converged upon by some later archosauromorphs, such as Macrocnemus (<https://www.nature.com/articles/s41598-020-68912-4>). **Noted**
Page 18 -
Lines 410-416 - Comparisons with pathologes in mesozoic or cenozoic diapsids would be ideal here, too. **Reference to** Resiz, R.R, Scott, D.M., Pynn, B.R., Modesto, S.P. Osteomyelitis in a Paleozoic reptile: ancient evidence for bacterial infection and its evolutionary significance. *Naturwissenschaften.* 2011;98(6):551-555.

Line 417 - Definitions for these conditions are so hyper-variable that a citation for the specific criteria would be helpful. **Done**

Page 21 -
Line 498 – Is there a specific way the acellularity of the tissue was determined? **Histological examination revealed it.**

Page 23 -
Line 541-542 – Does this refer specifically to the Permian of Oklahoma or something broader? Confusing as written.

**It refers to the broader early Permian.**

Page 25 -
Line 576-577 - You might cite an early amniote phylogeny that supports the suborbital fenestra as a diapsid character. **Cited**

Reisz, R.R. A diapsid reptile from the Pennsylvanian of Kansas. *Spec. Publ. Mus. of Nat. Hist., Univ. Kans*. 1981;7:1-74.

Carroll, R.L. 1981. Plesiosaur ancestors from the Upper Permian of Madagascar. Philosophical Transactions of the Royal Society of London, **293**(1066): 315–383.

Line 590 – Regarding this maxillary innervation character, you might note that more information on the plesiomorphic diapsid conditions and that of early lepidosauromorphs will be crucial to resolving this. Still, it's a VERY interesting hypothesis. **This is a comment, and I agree, more work is required.**

Page 26 -
Line 614 – Italicize O. mayorum. done

Page 28 -
Line 644 – Mosasaurs? done

Page 29 -
Line 668 – A few references to the new taxon possibly being a neodiapsid argue, in my opinion, for the inclusion of a phylogenetic analysis for the manuscript. I don't see it as an absolute necessity, but I think the neodiapsid references might best be removed otherwise. **Inclusion of the new taxon in Neodiapsida is confirmed by Schoch and Sues analysis, with addition of our taxon.**
Lines 673-675 – Odd to argue for a phylogenetic analysis in a paper that lacks such an analysis. You may indicate whether a follow-up is coming. removed
Line 674 – phylogenetic. done

Pages 30-33 -
There are many spelling errors, formatting issues, and italics problems through the citations. Please revise. done
