# Supplementary material for: An intriguing new diapsid reptile with evidence of mandibulo-dental pathology from the early Permian of Oklahoma revealed by neutron tomography
Source: PLoS One. 2022 Nov 30;17(11):e0276772. doi: 10.1371/journal.pone.0276772 (PMC9710763; doi:10.1371/journal.pone.0276772)
Supplement: Attachment — Submitted filename: Response to Reviewers.docx [file pone.0276772.s002.docx]

Additional Editor Comments :

L248 posterior-most  ? done, changed to “posterior-most”

L272: 5 to Five done

Ref: 32 the journal name is missing Done, added “Paleontol”

Added new reference to reference list as [47]

Maho T, Maho S, Scott DM, Reisz RR. Permian hypercarnivore suggests dental complexity among early amniotes. Nat. Commun. 2022;13:a4882.
